# Measures Undertaken in China to Avoid COVID-19 Infection: Internet-Based, Cross-Sectional Survey Study

**DOI:** 10.2196/18718

**Published:** 2020-05-12

**Authors:** Yu Huang, Qingqing Wu, Ping Wang, Yan Xu, Lei Wang, Yusui Zhao, Dingming Yao, Yue Xu, Qiaohong Lv, Shuiyang Xu

**Affiliations:** 1 Zhejiang Provincial Center for Disease Control and Prevention Hangzhou China; 2 Jiashan Prefectural Center for Disease Control and Prevention Jiashan China

**Keywords:** COVID-19, coronavirus disease, response, strategy, preventive measures, internet-based research, health QR code, outbreak, infectious disease, health education

## Abstract

**Background:**

In early 2020, over 80,000 cases of coronavirus disease (COVID-19) were confirmed in China. Public prevention and control measures, along with efforts from all sectors of society, were undertaken to control and eliminate disease transmission.

**Objective:**

This paper describes Chinese citizens’ response to the epidemic, the preventive measures they implemented to avoid being infected, and the public strategies that were carried out by the government, health workers, etc. We also discuss the efficacy of these measures in controlling the epidemic in China.

**Methods:**

Information on the responses and behaviors of Chinese citizens were collected through a cross-sectional, internet-based survey using Dingxiang Doctor’s public account on WeChat. Information on public strategies implemented by all sectors of society to control the epidemic and data on new COVID-19 cases were collected from the internet, mainly from government websites. Standard descriptive statistics and multivariate logistic regression analyses were conducted to analyze the data.

**Results:**

A total of 10,304 participants responded to the survey, with 10,198 valid responses; 74.1% (n=7557) were female and 25.9% (n=2641) were male. Overall, 98.2% (n=10,013) of participants paid high or very high attention to the epidemic, with WeChat being their main information source (n=9400, 92.2%). Over half the participants (n=5878, 57.7%) were confident that the epidemic could be curbed in China; 92.4% (n=9427) opened windows for ventilation more frequently than usual; 97.9% (n=9986) used masks in public; 95.7% (n=9759) avoided large crowds and stayed at home as much as possible; and 97.9% (n=9988) washed their hands more often than usual. Women were more likely to practice these behaviors than men (*P*<.001). With a series of strict public control measures, like nationwide health education campaigns, holiday extensions, the Examine and Approve Policy on the resumption of work, close management of working and living quarters, a health QR (Quick Response) code system, community screening, and social distancing policies, the number of new cases have decreased dramatically since February 12, 2020.

**Conclusions:**

The methods employed by Chinese citizens and authorities have effectively curtailed the spread of COVID-19, demonstrating that this pandemic can be brought under control as long as the right measures are taken.

## Introduction

In December 2019, an outbreak of pneumonia associated with the novel coronavirus disease (COVID-19) was reported in Wuhan, Hubei Province, China [1,2]. The timing of the COVID-19 outbreak, prior to the annual Chinese Lunar New Year holiday, coincided with people returning to their family homes, resulting in several billion person-trips made by residents and visitors [3]. In just 30 days, COVID-19 rapidly spread from a single city to the entire country, and from there to other countries around the world [4-7]. On January 30, 2020, the World Health Organization (WHO) declared the outbreak a public health emergency of international concern [8].

COVID-19 is a highly contagious disease. It mainly spreads from person to person through respiratory droplets, similar to the common cold and influenza viruses (ie, through face-to-face contact accompanied by a sneeze or cough). It can also be transmitted through contact with the secretions of infected individuals. The role of fecal–oral transmission is yet to be determined for COVID-19, but it was found to occur during the severe acute respiratory syndrome (SARS) outbreak [9]. The population is generally susceptible to the virus. Based on current epidemiological surveys, the latency period is usually 2-14 days (median 4 days), though longer cases have been noted [10]. COVID-19 is contagious during the latency period. At the time of submission of this paper, targeted antiviral drugs and vaccines were not yet available for COVID-19.

Under such a situation, it is important to provide the public with adequate information on risks and precautions, like the proper use of masks, frequent handwashing, and the avoidance of large gatherings, to control the outbreak. From January 20, 2020, when person-to-person transmission was confirmed and made public in China by Dr Zhong Nanshan [11], a renowned Chinese respiratory scientist, a large-scale, multilevel health education campaign was implemented in China. Information about COVID-19 spread swiftly on the internet, WeChat, microblogs, television, radio, and other media outlets. In rural areas, educational materials such as posters, bulletin boards, banners, and booklets were immediately provided to people with limited access to the internet. Core information included details on the epidemic situation, the government’s response to the epidemic, information on COVID-19, and self-protection strategies. Following the recommendations of health authorities, Chinese citizens put in place measures to protect themselves against COVID-19, such as staying at home as much as possible, limiting social contact, and wearing protective masks when in public.

During the early part of the outbreak, genetic analyses conducted in China revealed that the virus was similar to, but distinct from, severe acute respiratory syndrome coronavirus (SARS-CoV). China’s emergency management of SARS was heavily criticized in 2003 [12,13], and the Chinese government has improved its epidemic response capacity since then. National and local surveillance systems to prevent and control diseases were established and strengthened; the Field Epidemiology Training Program was initiated to increase the capacity of health workers at different levels; and laboratory capacity, collaboration, and communications with the WHO and the international scientific community were increased and strengthened [14]. The new and potentially serious outbreak of COVID-19 presented an opportunity to evaluate the efficacy of changes in China’s emergency management approach.

This paper aimed to describe Chinese citizens’ responses to the epidemic, the preventive measures they implemented to avoid being infected, and public strategies carried out by the government, health workers, etc. We also discuss the efficacy of these measures in controlling the epidemic in China.

## Methods

### Data Collection

Data collection for our study comprised two parts. First, information on personal responses and preventive measures to avoid being infected with COVID-19 were collected through a cross-sectional, internet-based survey. The questionnaire collected sociodemographic data (sex, age, occupation, education level, marriage, etc); sources used to obtain information on COVID-19; the level of attention paid to the epidemic; respondents’ confidence in the curbing of the outbreak in China; places that participants had visited during the COVID-19 epidemic period prior to the survey; and preventive measures taken to avoid infection. A 5-point Likert scale (ie, very low=1 to very high=5) was used to evaluate the participants’ confidence in curbing the outbreak by China and their level of attention to the COVID-19 epidemic. The questions about preventive measures were as follows:

1. What did you do to protect your families and friends?
2. Did you open windows for ventilation more frequently than usual?
3. Did you wear a mask in public?
4. Did you avoid large crowds and stay at home as much as possible?
5. Did you wash your hands more often than usual?
6. Under the following circumstance, do you wash your hands after touching public goods, toilet use, returning home, coughing/sneezing, or before eating?
7. Do you wash your hands with soap and running water in most cases?
8. Do you cover your coughs/sneezes with tissue or your bent elbow in most cases?

Questions about COVID-19 were based on the latest official report from the WHO, the Chinese Center for Disease Control and Prevention, and scientific literature. The original questionnaire was developed in Chinese by research group members from the Zhejiang Provincial Center for Disease Control and Prevention and refined over two rounds of Delphi method collaboration. A pilot study was conducted to verify its reliability.

Second, information on public strategies carried out by government, health workers, companies, etc, to protect citizens from being infected were sourced from official government websites, national and local health commission websites, national and local centers for disease control and prevention websites, and official papers [15-26]. Data on new COVID-19 cases were collected from the official website of the National Health Commission of the People’s Republic of China to describe the epidemic trend in China [15].

### Participant Recruitment

Participants were recruited via Dingxiang Doctor, a WeChat public account with 35 million users in China, used to disseminate health knowledge to the general population. A message stating “COVID-19, have you done enough to prevent it?” was created on the site, with a link to the questionnaire. Users who viewed the message could share it through the internet, allowing for news and information to spread quickly to many people. The study period was from January 31 to February 2, 2020.

The study was approved by the Ethics Committee at Zhejiang Provincial Center for Disease Control and Prevention. Informed consent was obtained from all participants before their information was collected.

### Statistical Analysis

Data were exported from Dingxiang Doctor to Microsoft Excel and analyzed using SPSS, version 19.0 (IBM Corporation). Standard descriptive statistics were used to summarize the data. Multivariate logistic regression analyses were conducted to explore differences in the practice of preventive behaviors between men and women. *P* values <.05 were considered statistically significant (two-sided). Epidemic trends were depicted visually and main public strategies used in China in response to COVID-19 were chronologically described.

## Results

### Sociodemographic Characteristics of Respondents

A total of 58,000 Dingxiang Doctor users visited the study page, 17.8% (n=10,304) of whom completed the questionnaire; 106 participants outside of China were excluded and 10,198 responses were included in the study. Table 1 presents participants’ sociodemographic characteristics.

Among the participants, 74.1% (n=7557) were female and 25.9% (n=2641) were male; 47.4 (n=4770) were single, divorced, or widowed; 52.6% (n=5364) were married. The proportion of participants in the age groups <30 years, 30-49 years, and ≥50 years were 55.4% (n=5653), 39.8% (n=4059), and 4.8% (n=486), respectively. Most participants had an undergraduate degree (n=7179, 70.4%), while participants with primary or lower, secondary, and postgraduate education comprised 5.1% (n=524), 10.8% (n=1105), and 13.6% (n=1390) of the sample, respectively. A total of 40.1% (n=4089) participants worked in the business and service industry, 17.7% (n=1809) worked in government institutions, 18.1% (n=1844) were students, 6.5% (n=666) were health workers, 7.3% (n=745) were housewives or househusbands, and 10.3% (n=1045) indicated “other” occupational statuses (retired, unemployed, etc).

**Table 1:** Survey respondents’ sociodemographic characteristics (N=10,198).

Characteristic		Respondents, n (%)
**Gender**		
	Male	2641 (25.9)
	Female	7557 (74.1)
**Age (years)**		
	<30	5653 (55.4)
	30-49	4059 (39.8)
	≥50	486 (4.8)
**Education**		
	Primary or less (≤9 years)	524 (5.1)
	Secondary (10-12 years)	1105 (10.8)
	Undergraduate (13-16 years)	7179 (70.4)
	Postgraduate (>16 years)	1390 (13.6)
**Occupation**		
	Staff of government institutions	1809 (17.7)
	Staff of business and service industry	4089 (40.1)
	Students	1844 (18.1)
	Health workers	666 (6.5)
	Housewives or househusbands	745 (7.3)
	Others (retired, unemployed, etc)	1045 (10.3)
**Marital status**		
	Single/divorced/widowed	4770 (47.4)
	Married	5364 (52.6)

### Personal Responses to the Epidemic and Preventive Measures Taken to Avoid Infection

#### Level of Attention to the COVID-19 Epidemic and Information Sources

Using a 5-point Likert scale, most participants indicated that they paid high attention to the epidemic. As seen in Table 2, 78.8% (n=8035) of participants expressed paying very high attention, and 19.4% (n=1978) paid high attention. In terms of information on COVID-19, WeChat was the participants’ main source of information (n=9400, 92.2%), followed by news and information apps (n=4529, 44.4%), microblogs (n=4154, 40.7%), television or radio (n=4042, 39.6%), family members, friends, or colleagues (n=2417, 23.7%), websites (n=2167, 21.3%), SMS (n=1110, 10.9%), short video apps (n=1606, 15.8%), community advocacy (n=1069, 10.5%), and paper-based media (n=671, 6.6%).

**Table 2:** Chinese citizens’ level of attention to the coronavirus disease (COVID-19) epidemic and their main information sources.

Variables		Total responses (N=10,198), n (%)
**How do you rate your attention to the COVID-19 epidemic?**		
	Very high	8035 (78.8)
	High	1978 (19.4)
	Neutral	170 (1.7)
	Low	11 (0.1)
	Very low	4 (0.04)
**Where did you get information on the epidemic?**		
	WeChat	9400 (92.2)
	News and information applications	4529 (44.4)
	Microblogs	4154 (40.7)
	Television or radio	4042 (39.6)
	Family members/friends/colleagues	2417 (23.7)
	Websites	2167 (21.3)
	Short video applications	1606 (15.8)
	SMS	1110 (10.9)
	Community advocacy	1069 (10.5)
	Paper media (newspaper, magazine, etc)	671 (6.6)

#### Preventive Measures to Avoid COVID-19 Infection

Most participants followed the recommendations of the public health authority. Table 3 shows that 92.4% (n=9427) of participants opened windows for ventilation more frequently than usual, 97.9% (n=9986) used masks in public, 95.7% (n=9759) avoided large crowds and stayed at home as much as possible, and 97.9% (n=9988) washed hands more often than usual. As for circumstances under which respondents felt inclined to wash their hands, 88.1% (n=8980) of participants washed their hands after touching public goods, 94.8% (n=9665) after toilet use, 91.5% (n=9331) after returning home, 66.5% (n=6782) after coughing or sneezing, and 87.5% (n=8923) before eating. In terms of how participants washed their hands, 86.7% used soap and running water in most cases. However, only 57.8% (n=5889) of participants covered their coughs and sneezes with tissue or a bent elbow. Furthermore, we found that most participants had tried to influence their families and friends: 84.8% (n=8652) told families and friends to avoid large gatherings, 88.6% (n=9037) asked them to stay at home as much as possible, 82.0% (n=8269) shared information on the epidemic with them, and 85.5% (n=8722) told them to wear a mask in public.

Gender differences existed in the practice of all preventive behaviors, with women being more compliant with all hygiene measures than men. Except for differences in “washing hands after coughing/sneezing or before eating” and “told friends and family to avoid large gatherings,” all differences were statistically significant (*P<*.05).

**Table 3:** Preventive measures taken by participants to avoid being infected with coronavirus disease (COVID-19), grouped by gender.

Variable		Gender		Total, n (%)	*P* value^a^
		Male, n (%)	Female, n (%)		
**What did you do to protect your family or friends?**					
	Told them to avoid large gatherings	2194 (83.1)	6458 (85.5)	8652 (84.8)	.07
	Persuaded them to stay at home as much as possible	2276 (86.2)	6761 (89.5)	9037 (88.6)	<.001
	Shared epidemic information with them	2062 (78.1)	6207 (82.1)	8269 (82.0)	<.001
	Told them to use a mask in public	2205 (83.5)	6517 (86.2)	8722 (85.5)	<.001
**Did you open windows for ventilation more frequently than usual?**					.003
	Yes	2394 (90.6)	7033 (93.1)	9427 (92.4)	
	No	247 (9.4)	524 (6.9)	771 (7.6)	
**Did you wear a mask in public?**					.001
	Yes	2563 (97.0)	7423 (98.2)	9986 (97.9)	
	No	78 (3.0)	134 (1.8)	212 (2.1)	
**Did you avoid large crowds and stayed at home as much as possible?**					<.001
	Yes	2488 (94.2)	7271 (96.2)	9759 (95.7)	
	No	153 (5.8)	286 (3.8)	439 (4.3)	
**Did you wash your hands more often than usual?**					<.001
	Yes	2557 (96.8)	7431 (98.3)	9988 (97.9)	
	No	84 (3.2)	126 (1.7)	210 (2.1)	
**Under the following circumstance, do you wash your hands?**					
	After touching public goods	2262 (85.7)	6718 (88.9)	8980 (88.1)	.001
	After toilet use	2424 (91.8)	7241 (95.8)	9665 (94.8)	<.001
	After returning home	2330 (88.2)	7001 (92.6)	9331 (91.5)	<.001
	After coughing/sneezing	1706 (64.6)	5076 (67.2)	6782 (66.5)	.06
	Before eating	2279 (86.3)	6644 (87.9)	8923 (87.5)	.11
**Do you wash your hands with soap and running water in most cases?**					<.001
	Yes	2196 (83.2)	6646 (87.9)	8842 (86.7)	
	No	445 (16.8)	911 (12.1)	1356 (13.3)	
**Do you cover your coughs/sneezes with tissue or a bent elbow in most cases?**					.01
	Yes	1451 (54.9)	4438 (58.7)	5889 (57.8)	
	No	1190 (45.1)	3119 (41.3)	4309 (42.3)	

^a^Multivariate logistic regression, adjusted for age, education level, occupation, marital status, and region.

#### Places Participants Had Visited During the COVID-19 Outbreak (Before the Survey)

From January 20, 2020, when person-to-person transmission was confirmed and made public in China, social distancing policies were advocated for among the public. As shown in Table 4, the most common place visited by participants before the survey were supermarkets or malls (n=5109, 50.1%); 26.9% (n=2648) of participants always stayed at home, 17.2% (n=1755) of participants went out to gather with friends or family members, 14.0% (n=1427) visited farmers’ markets, 11.0% (n=1120) used public transport, and 8.8% (n=898) went to their workplaces.

**Table 4:** Places that participants had been to during the coronavirus disease (COVID-19) epidemic period prior to taking the survey.

Place	Total responses (N=10,198), n (%)
Supermarket or shopping mall	5109 (50.1)
Always at home	2648 (26.0)
Gathering with friends or family members	1755 (17.2)
Farmer’s market	1427 (14.0)
Public transport areas	1120 (11.0)
Workplace	898 (8.8)

#### Participants’ Confidence in Curbing the COVID-19 Epidemic

Over half the participants were confident that the COVID-19 epidemic would be curbed in China, among whom 29.6% (n=3014) participants were strongly confident, and 28.1% (n=2864) were confident. A total of 30.1% (n=3073) participants were neutral, 10.0% (n=1022) lacked confidence, and 2.2% (n=225) strongly lacked confidence (Table 5).

**Table 5:** Participants’ confidence in curbing the COVID-19 epidemic in China.

Confidence level	Total responses (N=10,198), n (%)
Strongly confident	3014 (29.6)
Confident	2864 (28.1)
Neutral	3073 (30.1)
Lack of confidence	1022 (10.0)
Strong lack of confidence	225 (2.2)

### Main Public Strategies and Epidemic Trend of COVID-19 in China

Since January 20, 2020, a series of strategies were implemented to curb the spread of the virus to the wider community. Table 6 describes the main public strategies implemented by the government, health workers, factories, companies, etc, in response to the epidemic. In addition to active contact tracing, isolation and quarantine policies, a health education campaign, extension of the Chinese Lunar New Year holiday, social distancing policies, an Examine and Approve Policy on the resumption of work, close management of working and living spaces, and a health QR (Quick Response) code system, among others, were implemented.

**Figure 1:**
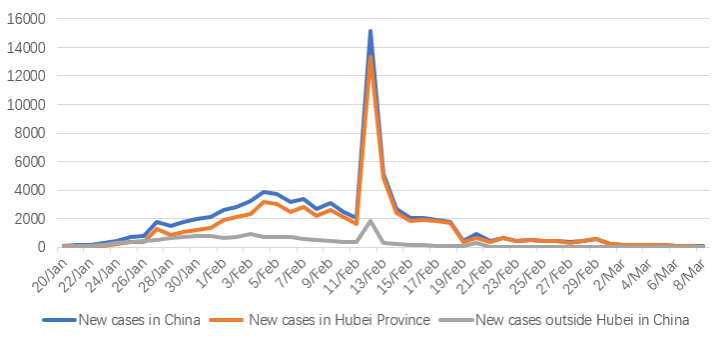
Daily new cases of coronavirus disease (COVID-19) in China since January 20, 2020.

Figure 1 shows the epidemic trend of COVID-19 in China. With the approach of the Lunar New Year holiday, mass population movement provided opportunity for the spread of COVID-19, and new cases emerged and increased in cities outside of Wuhan. With social distancing and a range of accompanying epidemic control measures, the number of daily new cases decreased continually after February 12.

**Table 6:** Main public strategies that responded to coronavirus disease 2019 (COVID-19) epidemic in China.

Date	Main strategies implemented by the government, health workers, factories, companies, and media
January 20	Person-to-person transmission is officially announced to the public. Nationwide health education campaign is initiated. People are encouraged to stay at home, avoid gatherings, wear protective masks when they need to move in public, etc.
January 23	The government extends Chinese Lunar New Year holiday. Museums, libraries, shopping malls, etc, are closed. Large public events are canceled or postponed. Chinese authorities place a lockdown on Wuhan, the epicenter of COVID-19, and traffic in Wuhan and cities across Hubei Province is restricted and monitored. Transportation is subsequently restricted at a national level.
January 24	The first batch of medical teams from outside Hubei arrive in Wuhan. More medical staff continue to arrive, totaling over 40,000 individuals. Chinese companies are ordered to build a 1000-bed hospital within 10 days. Work on a second facility with 1300 beds follows 2 days later. To plug the shortage of protective suits, masks, and other medical supplies, Chinese manufacturers from various industries are mobilized, including those that normally manufacture cars and cellphones.
January 27	The Examine and Approve Policy on resumption of work is initiated. Factories, companies, etc, begin to collect the travel history and health status of staff members.
February 3	Psychological service is provided nationally. A training program is set up on the National Health Commission website for continuing medical education [20] to share the latest knowledge on prevention, diagnosis, and treatment of COVID-19, to enhance the capacity of health workers.
February 4	Close management of communities, villages, and workplaces to curb COVID-19 begins in Hangzhou and is later implemented nationally. Community screening is initiated.
February 10	China urges efforts to ensure orderly resumption of work to provide sufficient material support for epidemic control. The health status of workers is examined and recorded before they resume work and monitored twice a day during work.
February 11	A health QR^a^ code system is developed by Alibaba Company, and implemented to control the spread of COVID-19 in Hangzhou. People traveling to Hangzhou must report their travel history and health conditions in advance online and are issued with green, yellow, or red QR codes, based on the information they had provided. A green code holder, rated as having little chance of being infected, can visit public areas and take public transport normally after taking their temperatures. Those with yellow and red codes, however, must be quarantined for 14 days and report their health information every day, or be sent to a hospital if necessary, before they may travel. Subsequently, the system is implemented nationally.
February 24	With the decrease in new cases, shopping malls, libraries, museums, etc, are reopened in succession.
February 28	The government publicly announces that the COVID-19 epidemic in Wuhan is controllable and is heading in a good direction.

^a^QR: Quick Response.

## Discussion

### Principal Findings

This is the first large-scale, nationwide study to report on the comprehensive preventive measures taken by Chinese citizens and strategies implemented by the government, health workers, factories, etc, to combat COVID-19 at a national level. We found that Chinese citizens responded quite well to the COVID-19 epidemic, by strictly following the recommendations of health authorities. The frequency of taking preventive measures by participants was high, with 92.4% of participants opening windows for ventilation more often than usual, 99.4% of participants using masks in public, 95.7% of participants avoiding large crowds and staying at home as much as possible, and 97.9% of participants washing hands more often than usual. However, responses varied among participants, and women showed higher compliance with all hygiene measures than men—a phenomenon that was also found in other studies [27-30]. Furthermore, fewer males than females participated in our survey (25% vs 75%); another internet-based study on Zika also had a similar gender distribution in terms of participants (30% male vs 70% female) [30]. It may be that women are generally more concerned about their health than men, so they are more willing to accept official recommendations and participate in health-related surveys.

Compared with measures taken to prevent the transmission of SARS in 2003 [30], we found a much higher frequency of masks usage (99.4% vs 68.7%), although we also found a lower frequency of covering one’s mouth when sneezing or coughing (57.8% vs 70.6%), and a lower frequency of hand washing after sneezing or coughing (66.5% vs 75.9%). The frequency of washing hands with soap was similar between participants of the two studies. Our findings suggest that although the health education campaign was quite effective in China, further education on practicing respiratory hygiene is needed, as sneezing and coughing are an important transmission route for COVID-19. Moreover, gender should be taken into consideration when planning and implementing health promotion and education programs.

WeChat, the largest standalone social media mobile app in China, was found to be one of the main sources of information on the COVID-19 epidemic for over 90% participants. In China, over 95% of adults own a mobile phone and over 1 billion access WeChat at least once a day. The app has become an integral part of Chinese daily life, with citizens using it to send messages, share updates, and access the latest news from the government, celebrities, enterprises, and so on [31]. Our study further confirmed that WeChat has become an ideal platform for delivering health-related information. Other studies from China also found WeChat to be an effective health behavior intervention platform [31-33].

Our findings showed that over 80% participants had tried to influence their families and friends by telling them to avoid large gatherings and stay at home as much as possible, sharing epidemic information with them, and telling them to use masks when necessary. This finding supports previous findings that participants who lived with their families were more likely to use a higher number of precautionary measures than those who did not live with their families during the SARS outbreak in 2003 [30].

Innovative measures combined with traditional strategies have facilitated China’s response to COVID-19. Traditional strategies, including active contact tracing, isolation and quarantine, a series of social distancing policies, and community containment were reinforced at a national level. China’s emergency management of SARS was heavily criticized in 2003 [12,13]; however, rapid and decisive strategies and the continuing decline of daily new cases of COVID-19 have earned favorable comment [14]. In addition to traditional measures, big data and mobile internet technologies were used to prevent the spread. On February 11, 2020, a unified health QR code system was promoted, first in Hangzhou, and then nationwide. The QR codes, generated with the mobile app Alipay, were based on users’ movements over a 14-day period, including whether users had been to virus-hit areas and had contact with confirmed or suspected cases. Instead of completing a health report form, Chinese citizens can now show the QR code when required; this is a much more time efficient approach to reducing the risk of virus transmission. It has also helped to facilitate traffic and speed up the resumption of work and production, ultimately promoting regional economic recovery [18]. Further, a training program was set up on the National Health Commission website for continuing medical education to share the latest knowledge on prevention, diagnosis, and treatment of COVID-19 to enhance the capacity of health workers around China [20]. Due to restrictions on gatherings during the epidemic and continuous deepening of knowledge on COVID-19, online education is an appropriate way to empower health workers.

### Limitations

This study has some limitations. First, selection bias might exist. Intrinsic limits, such as partial coverage of the population and missing responses from interviewees, are also present in our study, like other internet-based surveys [34,35]. Participants in our study were younger and more educated compared to the whole population. However, this allowed for the participation of a large group of geographically disparate participants at one discrete time point in a cost-effective and time-efficient manner. For population studies on infectious diseases, like SARS and COVID-19, internet-based surveys are the most appropriate method for data collection, since it avoids transmission of the virus during face-to-face investigation. Second, the number of daily new cases used to describe the trend of the COVID-19 epidemic in China is likely higher due to insufficient testing capacity and difficulties in identifying and counting mild and asymptomatic cases. However, the decline in new cases creates capacity for daily testing. Third, public strategies described in our study represented the main measures taken by the government, health workers, companies, and factories, and we may have missed some details due to restrictions on paper length.

### Conclusions

Our study provided a broad description of preventive measures taken by Chinese citizens to avoid being infected with the novel coronavirus and the main strategies implemented by the government, health workers, factories, companies, and media to protect citizens. Chinese citizens followed the hygiene recommendations of health authorities very well; however, further education on practicing respiratory hygiene is still needed in China. In addition to traditional response strategies, an innovative health QR code system based on big data and mobile internet technologies has helped to prevent the spread of COVID-19 and facilitate the resumption of work and production. Online education has enhanced the capacity of health workers on the prevention, diagnosis, and treatment of COVID-19 in a safe and efficient way. With daily decreases in the number of new cases, we conclude that Chinese measures to curb the spread of COVID-19 has been effective. Our findings suggest that an epidemic of COVID-19 can be brought under control as long as the right measures are taken.

## Abbreviations

COVID-19: coronavirus disease 2019
QR: Quick Response
SARS: severe acute respiratory syndrome
SARS-CoV: severe acute respiratory syndrome coronavirus
WHO: World Health Organization
